# American Citizens’ Views of an Ideal Pig Farm

**DOI:** 10.3390/ani7080064

**Published:** 2017-08-22

**Authors:** Patrycia Sato, Maria J. Hötzel, Marina A.G. von Keyserlingk

**Affiliations:** 1 Animal Welfare Program, 2357 Main Mall, Faculty of Land and Food Systems, University of British Columbia, Vancouver, BC V6T 1Z4, Canada; sato.patrycia@gmail.com; 2 Laboratório de Etologia Aplicada e Bem-Estar Animal, Departamento de Zootecnia e Desenvolvimento Rural, Universidade Federal de Santa Catarina, Florianópolis 88034-001, Brazil; maria.j.hotzel@ufsc.br

**Keywords:** animal welfare, attitude, business operation, consumer, ethics, naturalness

## Abstract

**Simple Summary:**

The public, who also make up the largest proportion of consumers of animal products, often criticize farm animal industries in regards to their care and handling of farm animals. The U.S. swine industry has not been exempt from such criticisms. The aim of this study was to explore the views of the people not affiliated with the swine industry on what they perceived to be the ideal pig/pork farm, and their associated reasons. Through an online survey, participants were invited to respond to the following open-ended question: “What do you consider to be an ideal pig/pork farm and why are these characteristics important to you?”. Respondents considered animal care, profitability, farm size, compliance with sanitary, environmental rules and regulations, farm cleanliness and sanitary standards, and workers’ rights and welfare important, but also raised concerns relating to pigs’ quality of life including space to move, feeding, contact with outdoors or nature, absence of pain, suffering and mistreatment. Perspectives were also raised regarding the ideal farm as a profitable business operation, clean, and with optimal sanitary conditions. Respondents also emphasized naturalness, frequently stating that pigs should have access to the outdoors, and rejected the use of hormones, antibiotics, and other chemicals for the purposes of increasing production.

**Abstract:**

Food animal production practices are often cited as having negative animal welfare consequences. The U.S. swine industry has not been exempt from such criticisms. Little is known, however, about how lay citizens who are not actively engaged in agricultural discussions, think about swine production. Thus, the aim of this study was to explore the views of people not affiliated with the swine industry on what they perceived to be the ideal pig/pork farm, and their associated reasons. Through an online survey, participants were invited to respond to the following open-ended question: “What do you consider to be an ideal pig/pork farm and why are these characteristics important to you?”. Generally respondents considered animal welfare (e.g., space, freedom to move, and humane treatment), respondents considered the business operation role important for pork production (e.g., profitability, compliance with sanitary, environmental rules and regulations, and workers′ rights), and naturalness (e.g., natural feeding, behaviours and life) important for pork production. Concerns relating to pigs’ quality of life included space to move, feeding, contact with outdoors or nature, absence of pain, suffering and mistreatment. Perspectives were also raised regarding the ideal farm as a profitable business operation, clean, and with optimal sanitary conditions. Respondents also emphasized naturalness, frequently stating that pigs should have access to the outdoors, and rejected the use of hormones, antibiotics, and other chemicals for the purposes of increasing production. In summary, the findings of this study suggest that the U.S. swine industry should strive to adopt animal management practices that resonate with societal values, such as ensuring humane treatment, and the failure to do so could risk the sustainability of the swine industry.

## 1. Introduction

As the United States (U.S.) came out of the Great Depression food shortages were addressed by improving efficiencies of food animal production, resulting in dramatic changes in the way farm animals were housed [1]. Confinement indoor housing methods became the norm, particularly after the Second World War, for poultry, pigs, veal calves, and laying hens [2]. Over the last century much of the global pork industry has transitioned from small, outdoor herds, to large, intensive indoor systems [3]. Currently, the U.S. is the world’s third-largest producer and consumer of pork products, representing an important activity in the agricultural economy, with exports averaging approximately 20% of commercial production [4].

However, intensive pork production has come under scrutiny from stakeholders external to the industry; these concerns are focused primarily on the quality of life afforded to pigs [5,6], and have resulted in dramatic changes in how pigs are housed and cared for in the European Union [7]. However, despite these changes, producer-led changes in other parts of the world have been slow, with many farmers defending their current practices, often arguing that urban citizens are ignorant of farm practices, and therefore should not be consulted [5]. More recently, retailers and processors have become globally active participants in the discussion of how farm animals should be cared for on farms (see review [8]); these initiatives have no doubt increased public awareness and ensuing concerns for farm animal welfare in the U.S. [9].

Given the continued pressure placed on U.S. animal agriculture by stakeholders external to the pig industry (e.g., retailers and animal advocates), it is important to understand what lay individuals believe are ideal characteristics that result in a good life for pigs on U.S. farms. Although there is vast literature on public perceptions regarding pig welfare in European countries (see review by [10]), less is known regarding citizens of other countries [11], including the U.S. [12,13,14]. Thus, the aim of this study was to explore the views of U.S. citizens with little experience with the U.S. pork industry on what they perceived to be the ideal pig farm and their associated reasons. In a survey conducted in Denmark, Lassen et al. [15] found that, when talking about pork, the interviewees characteristically limited their remarks to price and what can be described as the material quality of pork. Thus, an additional objective of this study was to assess whether there would be differences in response patterns by adopting the expression “pig farming” or “pork farming”, considering that maybe the latter could dispel aspects related to the animals as sentient beings [16].

## 2. Materials and Methods

This survey was completely anonymous and approved by the University of British Columbia Behavioural Research Ethics Board (H13-01466). Data were collected via the online platform Fluid Surveys (Fluid Surveys, http://fluidsurveys.com/), and participants were recruited via Amazon’s Mechanical Turk (MTurk, Provo, UT). Several studies have assessed MTurk and shown that this approach results in high-quality and reliable data (e.g., [17,18,19]). This survey is a convenience sample, and thus is not meant to be representative of the entire US population. Upon completion the participants were paid (U.S. $0.50). The survey was launched on 10 April 2015. The cohort consisted of 200 U.S. residents, who were recruited within 48 h of launching the survey. The goal of this type of qualitative work is not to focus on frequencies and relative importance of the arising themes per se, but eliciting all possible views. This can be done by increasing the number of participants sampled until such time no new views arise, normally referred to as data saturation. The work that arises from these types of studies can then be used to inform future work that is then based on representative sampling, which can then be used to make strong inferences of the views of a population.

Upon entering the on line platform, participants were given the following information before taking the survey, which was adapted from [20]: “Take a short survey asking your opinion of pig farms. We want to know what characteristics you think make the ‘ideal’ pig farm”. Due to the possibility of these different interpretations, we used both terms “pig farm” and “pork farm”, and thus randomized these two treatments given to participants. Participants were then asked to give written informed consent and then provided access to a single open-ended question: “*What do you consider to be an ideal (pig, pork) farm and why are these characteristics important to you?*”. They were free to express any aspects they felt were important. Participants were then asked 11 multiple-choice demographic questions after answering the study question (see Supplementary Material).

### Survey Analysis

Open-ended responses (on average 65 words) were analyzed based on methods described initially by Huberman and Miles [21]: *Data reduction* (information is coded to find themes), *data display* (organization of the information permitting to reach conclusions), and *conclusion drawing and verification* (noting of patterns and themes and using confirmatory tactics such as consistency between coders).

Applied thematic analysis was the analytical framework used in this study. Three trained evaluators blind to demographic information, initially independently examined a subset of the responses, that included inductive, data-driven coding that was used to develop themes that answered our research question—What is an ideal pig/pork farm? Then the first author did the analyses, which included initial line-by-line coding, a process of defining and labeling segments of data with words or short phrases [22]. Inter-coder agreement, a process whereby another researcher analyzes the same data and compares and discusses the results [22] was undertaken, and any discrepancies were discussed among the three authors.

Three readers compared results and reconciled any discrepancies before the final analysis was undertaken. The thematic analysis of the responses identified six primary themes (Table 1). The main themes arose from the responses rather than being determined *a priori*. The quotes were selected to represent examples of excerpts within responses that had been classified under a given code within each theme; preference was given to statements that contained a concept shared by many responses, or those that better expressed a given concept.

## 3. Results

Demographic data are presented on Table 2. Of the two hundred responses, 94 were related to an ideal pig farm, and 105 to an ideal pork farm. Respondents were from 30 U.S. States (no responses were obtained from Alaska, Arkansas, Delaware, Idaho, Indiana, Iowa, Kansas, Kentucky, Maine, Maryland, Minnesota, Mississippi, Montana, New Mexico, North Dakota, Rhode Island, South Dakota, Utah, West Virginia, and Wyoming). Overall our participants were largely from the millennial generation, ranging in age from 25–34, with the majority holding at least a Bachelor’s degree. The participants declared themselves largely unfamiliar with pork production; 86.2% of respondents from the pig cohort (C1), and 85.7% of respondents from the pork cohort (C2) self reported as not being involved with pig farming. We noted no clear differences in the responses between both cohorts; respondents of both cohorts understood a pig or pork farm as a commercial unit that produced pork for human consumption, resulting in similar concerns raised by both cohorts (Table 1). We therefore report the results together, but when providing a quote we clarify whether it was a respondent from the C1 (pig) or C2 (pork) cohort.

Results of the qualitative responses are described per main themes, which are described per order of prevalence.

### 3.1. Animal Welfare

Animal welfare was the most mentioned theme: 74% of the respondents addressed it when describing or justifying the features they considered important or essential in an ideal farm. The majority of respondents focused their responses in terms of concerns relating to animal welfare, including space to move, feeding, contact with outdoors or nature, absence of pain, suffering, and mistreatment; a number of references in relation to animal sentience were also made, with participants using positive terms such as “happiness” and “intelligence”. The ethical background underpinning animal welfare concerns was clearly present in a large proportion of the respondents in that they used words such as “respect”, “decency”, “dignity”, and most notably, “humane” to refer to animal treatment.

#### 3.1.1. Space and Animal Freedom

The most frequently noted characteristic of the *ideal pig farm* was space*,* mentioned by 44% of the respondents—reflecting concerns about the animals’ housing. For example, respondents stated that in an ideal pig farm: “*The pigs have room to roam and aren’t trapped in pens until they are fat enough to slaughter.”* (Resp. C1 1); “*It would be a farm where the pigs are kept in a sanitary environment, and not overcrowded.*” (Resp. C1 199); *“Where animals are given space to roam and not piled on top of one another.*” (Resp. C2 146).

In the context of space, some respondents (9%) criticized the confined system: “*The use of cages should not be permitted*” (Resp. C2 125); “*Plenty of room for the pigs! Those I’ve seen are normally too small and too cramped with far too many pigs in such a tiny pen.*” (Resp. C1 81); “*I think an ideal pork farm should be spacious. I read a lot about the horrid conditions that pigs are kept in such as severe overcrowding. I would want the pigs to have plenty of room to move around.*” (Resp. C2 156). A few respondents (4%) seemed to consider confined housing acceptable, as long it provides sufficient space: “*They should be able to walk around on grass in penned areas and be able to move freely in their pens*” (Resp. C1 56). “*There should be plenty of space for them in their pen, for when they breed and needed for the piglets to grow.*” (Resp. C1 87).

Another frequently mentioned feature (by 20% of the respondents) was the need for an outdoor area for pigs where they could move around, interact with each other and perform natural behaviors: “*The pigs will have access to the outdoors, to grass, to vegetation*” (Resp. C1 8); “*I think the pigs should have enough room to move about and socialize with other pigs while they are there. They should have access to outdoors as well as shelter. Mud is mandatory*” (Resp. C2 147); “*The ideal pork farm to me would be a large designated area where the pigs are free to roam and forage for food as they would normally and naturally.*” (Resp. C2 156). Interestingly 12 people (6%) mentioned the term “free range”.

Some people also acknowledged pigs as intelligent animals, emphasizing the importance of the facilities for a happy life. For example, one participant described that at the ideal pig farm animals “*Are given plenty of space and clean living conditions. Pigs are intelligent animals and deserve a good life*” (Resp. C1 92); “*The ideal pig farm would probably be relatively cruelty free as pigs are relatively intelligent animals and I see no point in them suffering unduly before they are killed and butchered*” (Resp. C1 69).

Some respondents related offering more space and free-range housing to meat quality: “*I think proper living space for the pigs is the most important characteristic, since pigs that are able to roam and graze are happier, and provide better tasting pork*” (Resp. C1 17); “*An ideal pig farm is a farm that has wide open space for pigs to roam and feed. This is important as I feel keeping pigs cramped, enclosed space will help spread disease and infections. The pigs will also produce better meat as they are healthier and less stressed*” (Resp. C1 19). However, it was clear that many associated more space with other farm features that they believed to improve meat quality: “*The ideal pork farm is one that is small enough to have all the pork be free-range and slop fed. The pork farm has nice handlers, and kills the pigs humanely. It’s important that the meat I eat be happy, and happy pigs come from being treated well.*” (Resp. C2 191); “*The pigs would have humane lives before slaughter, plenty of room to roam and be fed organic, sustainable foods in order to grow. This makes for a better meat product, and something that is healthier for the general public.*” (Resp. C2 186).

Respondents also referred to the need for veterinary care to be provided to the animals, such as: “*They should have adequate veterinary care*” (Resp. C1 56). Animal health was also frequently associated with meat quality; such as “*the pigs need to be raised in a healthy manner to produce the best pork to eat*” (Resp. C2 156).

#### 3.1.2. Salient Terms and Features Used in Reference to Pigs’ Welfare

Some terms used by a high proportion of the respondents were:

(1) “Humane” (41%; e.g., “*The one of utmost importance is humane treatment of the animals*”, (Resp. C2 154); “*A farm that is productive, but humane to the animals*”, (Resp. C2 139)).
(2) For 36% of the respondents, pigs should be “well fed” (including appropriate type and amount of feed, grass-fed or natural, free of chemical residues, or organic feed): *All pigs should be well fed, appropriate medical care, and lead a relatively enjoyable life until it’s time for them to become food*. (Resp. C2 147). Similarly, one participant stated “*an ideal farm should have the animals roaming relatively freely and feeding on natural foods like grass.*” (Resp. C2 189).
(3) Specific objection to “animal mistreatment”, and calls for good animal treatment were mentioned by 21% of the participants. The quality of treatment of animals was raised regarding slaughter (“*The pigs would be treated humanely and would be slaughtered in a honest and safe manner*”, (Resp. C1 66), general animal care (“*A farm where all the pigs are well cared for and treated humanely*”, (Resp. C1 43), human handling (“*The pig should be treated in an ethical manner as well, they should not be beaten or treated in a manner that is inhumane*”, (Resp. C2 194), and animal health (“*This* (good care) *is important because diseases and sickness can spread more easily where the pigs are forced to group together too tightly.*”, (Resp. C2 158).
(4) “Free” or “freedom” (16%; e.g., “*Somewhere where the pigs are allowed outside to live freely and roam and play.*” (Resp. C1 36); “*An ideal pig farm would be one where pigs have a lot of space to move around and plenty of food. These are important because we should give pigs freedom for their lives and not limit things for them*”, (Resp. C1 91)).
(5) Overall, the “absence of pain and suffering” was mentioned by 12% of the respondents: “*It is important to know that the animals did not suffer*” (Resp. C2 104); “*Well I’d like the pigs to lead lives of minimal pain.*” (Resp. C2 109); “*I would also want the farm to slaughter them in the most humane method possible to avoid as much pain as possible*” (Resp. C1 61); “*They would get some toys or something to enjoy life. Mother pigs would be able to rear their piglets. Piglets wouldn’t be castrated or teeth clipped without some form of pain killer*” (Resp. C1 21).
(6) “Happy pig” (10%; e.g., “*My ideal pig farm has happy pigs. They are able to roam and exercise and eat healthy foods. They live a long, full life before being butchered and are treated with the utmost respect*” (Resp. C1 34).

### 3.2. Business Operation

The second most mentioned theme was the business operation (43%), which included a variety of opinions concerning cleanliness and optimal sanitary conditions (21%), and production efficiency/profitability (11%).

Many respondents pointed out cleanliness of the pigs’ environment as an important feature associated with animal health conditions and, consequently, with human health: “*The facility is well maintained and clean*” (Resp. C1 64); “*It is important that the farm is clean so the pigs don’t become ill and become contaminated when they are slaughtered*” (Resp. C2 102). Some of the respondents also extended their responses to include good sanitary conditions “*the ideal farm has safe practices that lead to food being produced that is safe for human consumption*” (Resp. C2 116); “*An ideal pork farm would have a healthy environment for the livestock and wouldn’t use unnecessary antibiotics, etc.*” (Resp. C2 130).

For some, profitability production efficiency and low cost production were essential for the business, for example: “*The main objective of farm plan is to obtain maximum returns*” (Resp. C2 176); “*Another aspect of a perfect pig farm is the use of technology and automation. This will make the farm more efficient and more likely to keep the farm profitable*” (Resp. C1 19); “*The ideal pork farm will have a cost efficient operation. It will have a good HR department that paints a picture of happy hogs, but in reality the bottom dollar is important*” (Resp. C2 160).

Other aspects that respondents (5%) mentioned in relation to the business operation were that a pig farm should follow rules and regulations: “*An ideal pig farm would be a sanitary livestock facility that was constructed, maintained and utilized in a state and federally approved manner, where the workers are credentialed and properly trained in the proper, humane treatment of livestock. The equipment would pass all safety inspections and follow all approved operational regulations. The pigs would be feed to proper nutrients as outlined by the respective governmental agency. The farm would be regularly inspected and all inspection results posted in a public manner*” (Resp. C1 22); “*One that complies with all humane animal treatment regulations*”, (Resp. C1 15)*.* Additionally, the facility will train, respect and value its workers (4%), produce at low cost (3%), be located away from urban areas (2%, e.g., “*The ideal pig farm would probably be remote so others don’t suffer from the smell, laid out well for maximum efficiency*”, (Resp. C1 69)), produce locally (1%), and make good use of technology (1%, e.g., “*Another aspect of a perfect pig farm is the use of technology and automation. This will make the farm more efficient and more likely to keep the farm profitable*”, (Resp. C1 19)).

A few mentioned farm size, some stating that the farm should be small or “not too big” (3%) while others, stated that it should be big (3%). A few said an ideal pig farm should be family run (3%), e.g., “*A family run farm. They will put more into keeping the animals well since it means more to them money wise as well as personally instead of only bottom line for a big company*” (Resp. C2 98).

For a few respondents production related aspects were the only concern, for example: “*Organization, efficiency, and cleanliness is pretty much the main goals in my mind.*” (Resp. C2 164); “*The ideal one would have a giant air filtration system around so the stink doesn’t get out. Those things smell horrible when you drive by them. I don’t really care about the “quality of life for a pig”, it’s food to me.*” (Resp. C2 192).

### 3.3. Naturalness

Respondents (27%) mentioned naturalness, referring to natural feeding, natural behaviors, natural lives, natural farming, and nature. Many people expressed the wish that these animals were fed only with natural products: “*They would consume natural foods, and as a result of a mostly natural lifestyle they would be healthier*” (Resp. C2 123); *Pigs with enough space to run around freely, fed on a vegetarian diet and not injected with hormones or antibiotics. I think these are all important to ensure quality product that won’t harm people eating it.* (Resp. C2 131); *A farm where pigs can roam free and not be confined, where they are allowed to be outside or inside at any time, and where they can eat a variety of food that it’s natural for them to eat.*” (Resp. C1 7).

Others referred to the life of animals (“*One where the pigs are living a more natural life*”, (Resp. C1 15)), or more specifically to the ability of pigs to express their natural behaviors: “*Pigs would have mud to rest and play in*”*.* (Resp. C2 108*);* “*The females would be allowed to care for her offspring until such a time that separation would be natural*” (Resp. C2 120). Some expressed these preferences in terms of “natural farming”: “*An ideal pig farm should have a large amount of land/space for the pigs to roam, and a proper place for them to stay in the barns. They should be given a healthy diet, and shouldn’t be fed hormones for growth. All natural is important to me because I try to encourage the use of all natural farming methods.*” (Resp. C1 52); “*The ideal pig farm would be based on principles of sustainability and harmony with nature*”. (Resp. C1 39).

### 3.4. Ethical Considerations and Recognition of Trade-Offs

When conveying their opinions approximately 25% of the respondents provided their views from an ethical perspective, for example: “*I believe that the ideal pork farm is clean and that the animals are treated humanely. This is important to me because it is in line with my ideals and morals*” (Resp. C2 155). Some (10%) used terms like “decent” or “decency”, “respect”, and “dignity”: “*These characteristics are important to me because I believe that if they are going to be slaughtered for human benefit, we owe it to them to give them a decent life*”*.* (Resp. C1 32); “*I like bacon, and I feel better about eating bacon from places where the animals were treated at least decently before being killed for bacon consumption.*” (Resp. C2 199); “*First and foremost would be humane treatment—pigs that can go outside, sows that aren′t cooped up in those god awful (sic) farrowing crates, adequate space per animal, animals treated with kindness and dignity and provided with medical care when necessary and adequate and nutritious feed, no overbreeding*” (Resp. C2 106).

Some respondents stated that they eat pork and support pork production before emphasizing that they expect ethical treatment of the animals, for example: “*I believe that pigs, although it is acceptable to eat them, should be treated with respect while they are alive.*” (Resp. C2 119); “*An ideal pork farm is where pigs are treated properly and are not abused in any way. They are fed properly with good feed and their living conditions are good. This means a healthy environment for them to live, play, and roam. These characteristics are important to me because I think animals deserve to be treated properly. They are living and breathing so despite the fact that many end up as food, they don’t deserve any less while they’re alive.*” (Resp. C2 114).

Some identified a need for a balance between good practices, profitability, and meat quality, but also highlighted the importance of meeting government standards: “*An ideal pork farm is one that maximizes yield without causing the animals undue harm or compromises food safety*” (Resp. C2 165); “*The ideal pig farm should balance the animals’ welfare with the need to keep the meat sanitary*”. (Resp. C1 57); “*The entire process must comply with all state, local, and federal laws. The business must be as transparent as possible, to demonstrate to the public that they are doing everything that their industry considers to be important…the process must be efficient. Just because the company should care for the welfare of the animals doesn’t mean that they should be wasteful in spending or create a product that is incredibly expensive. They should streamline their operations and follow a business plan in order to keep costs low for the consumers*” (Resp. C2 151).

Others argued that keeping production costs low should be secondary to food safety, animal welfare, or environmental sustainability:

“*I would consider a pork farm to have enough room to allow the pigs to maintain a healthy lifestyle. This will also ensure that the pigs would have a less of a chance to pass on sicknesses to each other. I understand that this would raise the cost of manufacturing, but this price increase would be valuable to both the pig farm, and to the people who consume the product.*”

(Resp. C2 190)

“*I’d rather pay a little more money for pork knowing these animals were treated well and fed well.*”

(Resp. C2 146)

“*An ideal pig farm goes above and beyond by making sure that the pigs are raised in as good environment. It is important that they are well fed and have space to move around. The pig farm would not place such a high priority in maximizing profits. These characteristics are important because it is the decent thing to do.*”

(Resp. C1 13)

“*Humane treatment of the animals. They should be treated to a normal, natural life and not overcrowded and confined in their own waste until they are ready to be slaughtered. Less production would be a fair price to pay for humane treatment.*”

(Resp. C1 12)

“*A non-polluting, environmentally friendly farm where the animals are treated as kindly as possible and are dispatched as painlessly as possible. I don’t think profitability can justify cruelty. No business has a right to contaminate the community or its common resources.*”

(Resp. C1 24)

However, a few statements dismissed any farm goals pertaining to animal welfare, instead favoring productivity, for example: “*As cruel as it might be I’d consider the best pig farm to be one that can stuff as many pigs into as small a space as possible while maintaining decent cleanliness. The higher the efficiency, the lower the cost for me*” (Resp. C1 70); “*Whatever gets the pork to me fastest and cheapest*” (Resp. C2 148).

### 3.5. Overuse of Antibiotics, Hormones, and “Chemical Residues”

Some people (19%) described the ideal farm from the perspective that the farmers should engage in the rational use of chemicals: “*An ideal pork farm would have humanely treated pigs who are fed organically. It′s important that my meat sources are as humane as possible with as few chemicals as possible*” (Resp. C2 183); *“Antibiotics should be used only when needed”* (Resp. C2 196); but others were less tolerant regarding the use of these substances: “*The animals should not be getting antibiotics or hormones*” (Resp. C2 189); “*I consider an ideal pork farm to be organic. Meaning, the pigs are not injected with steroids, hormones, or antibiotics, and the feed they are given is not just slop, but rather decent non-pesticide soaked leftovers and feed*” (Resp. C2 141); “*It would practice sustainable and GMO free farming practices free of hormones*” (Resp. C2 187).

### 3.6. Impact on the Environment

A relatively small proportion of respondents discussed environmental impacts of pig production systems, suggesting that at least for these survey respondents this was likely not a primary concern. Some participants raised the issue of pollution generated by livestock and argued that manure management, in addition to the rational use of resources, such as water, should be addressed. In total, 13% mentioned some feature related to this theme, with 6% using the term “environmentally friendly”: “*It would have good drainage and be low in pollution. The owner would be mindful of best environmental practices for both air and water quality*” (Resp. C2 184); “*and a way to properly treat waste from the farm*” (Resp. C1 60); “*Acceptably low environmental impact is important because there is little sense in spoiling aquifers and other groundwater solely for the production of pig products; maintaining a potable water supply is far more important than maintaining a pork supply in the long run. As such, the pig farm must balance the need for pork against the weightier need for water*” (Resp. C1 53); “*I would say that the ideal pig farm is very sustainable meaning that the water use of the entire farm is kept to an absolute minimum*” (Resp. C1 18).

## 4. Discussion

Overall respondents described a pig or pork farm as an animal production unit that produces the pork that they eat, and from this perspective they discussed what conditions they considered important for pork production, such as animal care, profitability, farm size, compliancy with sanitary, environmental rules and regulations, farm cleanliness and sanitary standards, and workers’ rights and welfare. As our main objective was to identify important concerns regarding pig farming by individuals living in the U.S., we did not expect participants to rank the importance they attribute to each feature they mentioned. So, although more participants expressed concerns about pig welfare than any other issue, this does not mean that this is their first priority, or even that this influences their purchasing habits.

Space, largely associated with animals′ freedom to move and the ability to perform innate natural behaviors, was the main theme addressed by the respondents. Many described their desire for housing systems that are not overcrowded and that do not limit restriction of movement. Restriction of space has been shown to be a main pig welfare concern for European [23] and U.S. [12] citizens. Other studies have also reported that space, especially in relation to freedom of movement, is a major concern in current pig production systems (e.g., Germany [24], China [25], Brazil [26]). For the U.S. citizens who responded to our survey, it is possible that the salience of this concern reflects, at least in part, the effect of campaigns related to ballot initiatives in several U.S. states in recent years [27,28]. Alternatively, it may be argued that the animal protection organizations that led these initiatives recognized space and freedom as primary social values associated with livestock welfare, giving priority to this issue in their campaigns, which may have influenced some of the participants.

Providing pigs with access to the outdoors is highly valued, and inherently associated with higher animal welfare standards by European citizens [15,29,30]. In the current survey, providing pigs access to the outdoors or free-range housing was mentioned as a desired characteristic by fewer than 40% of the participants; but when it was raised it was almost always in response to concerns about the space provided to the animals, suggesting that lack of space and overcrowding represents a greater concern for our participants than a confined housing system per se. We see provision of more space as a possible opportunity for the swine industry, given that attempts to improve confined systems to comply with society’s expectations should lead to possible increased acceptance. For instance, Vanhonacker et al. [23] also concluded that the animal welfare image among the public in the case of confined pigs may benefit from providing farm animals with more space.

Other animal welfare related topics mentioned by respondents included the absence of pain and suffering, naturalness, and especially humane treatment. Accordingly, in a systematic review of the literature, Clark et al. [31] concluded that naturalness and humane treatment are the two top consumer concerns associated with farm animal welfare. In our study, slaughter was mentioned by 18% of the respondents, many demanding that it be humane, while others were more specific referring to wanting assurance that it occurs in the absence of pain and suffering. Two explanations suggested by McKendree et al. [12] for this elevated concern about slaughter in their survey with U.S. citizens (exposure to videos in the media showing mistreatment of animals at slaughterhouses and an inherent discomfort with killing animals for food consumption) were confirmed by statements made by respondents in our survey. Other important issues shown to reduce the welfare of pigs, including transportation [32] and tail docking [33] were not raised by our participants—and castration [34] was mentioned only by one participant. This could be an indication of a lack of awareness and may also reflect the focus on slaughter and restriction of movement (housing) by animal advocacy groups (and in turn the media) [12].

To our surprise, in contrast with other studies that discuss farm size as being a major concern identified by lay citizens, very few respondents raised this issue in our study. For example, large-scale industrial production has been heavily criticized in Europe [30,35]. In contrast to the Europeans, Chinese urban citizens seem to prefer industrial farms [36,37], a response that was echoed by some Brazilian consumers [38,39]. The preference for large industrial farms in some countries, particularly in emerging economies, by pork consumers may be associated with the perception that sanitary conditions and food safety standards are higher on these types of farms [36,37,40]. In the current survey, cleanliness and sanitary aspects were also highly valued as desired features of an ideal pig farm. Interestingly, consumer attitude surveys in general indicate that, even though consumers from different countries value animal welfare, food safety is the highest-ranking attribute mentioned by survey participants [13,41,42,43]. However, despite interview based surveys reporting that food safety and animal welfare concerns are highly valued, others have reported that these values are not reflected in consumer-purchasing behavior, as point-of-sale price remains a high priority [10,35,44].

Some participants considered it important to abolish the use of chemicals in pigs’ feed, e.g., antibiotics, hormones, and pesticides, a position likely reflecting concerns regarding a perceived overuse of antibiotics, hormones, and other chemicals for animal production purposes, in both developed [24,45] and developing countries [25,46,47]. This finding was also in agreement in studies covering other livestock species [20,48,49], where, collectively, respondents raised concerns about animals receiving natural, healthy, or organic feed, indicating a growing concern about this topic. In the same context, experimental work has focused on the intensive housing systems (and the associated conditions for pigs) and the overuse of antibiotics, hormones, and other similar products and their associated effects on food safety and meat quality [10,24,25,30].

Intensification of livestock production has been associated with negative effects on the environment, rural populations, biodiversity, and farm animal welfare [2,50], resulting in intense criticisms from social, animal, and environmental protection movements. Some participants in our study stated that farms should be located far from cities to avoid pollution, and mentioned the issue of smell coming from pig operations, echoing other research from the U.S. [14]. However, only a few participants discussed environmental impacts of pig farming or agriculture sustainability in a broader context, suggesting that the U.S. public has been largely absent from agriculture policy discussions [51,52,53]. One argument frequently used to defend the intensification of livestock production are the tremendous efficacies that can be realized in these types of systems, thus allowing for increases in the amount of food animal products needed to meet the perceived demand for food for the projected 9 billion global population by 2050 [54]. However, some have argued that large intensive animal production systems will be less well accepted despite being associated with practices that are viewed to be more sustainable, including reduced consumption of animal products and use of technologies [55,56,57]. However, others have shown that people do not seem willing to eat less meat or accept meat substitutes such as artificial meat [58,59,60]. It has also been suggested that equity, access, and distribution should be greater priorities than increased production [61]. However, in some countries policy discussions of this nature have only recently included animal welfare trade-offs [56,62]. Our study provides evidence that, at least for the respondents in our study, farm animal welfare and food safety are primary concerns and thus both should be given due consideration. We suggest greater efforts by U.S. policy makers to include the lay public’s views and expectations in these types of discussions. Failure to address this gap between farm practices, policy discussion, and societal expectations (see also von Keyserlingk, et al. [63]), could potentially threaten the long term sustainability of the U.S. pig industry.

Respondents highlighted linkages between different features that they considered necessary for an ideal pig/pork farm. For example, they linked animal welfare with economics and meat quality, meat quality with human health or nutrition, naturalness with animal welfare, and production systems with environmental sustainability. Several respondents also stated that reducing cost of production did not justify what they considered animal “abuse”. In some cases respondents stated their position by relaying perceived trade-offs between their preferences and other potentially competing factors. However, they never went beyond two-way relations, in other words they appeared to be largely unaware of the complexity associated with agriculture sustainability [64,65]. This may explain, at least in part, the disconnect between different stakeholders regarding animal agriculture, including the conception of animal welfare [5,66,67]. Farmers have argued that the lay public lacks information and familiarity with pig farming issues, and therefore have unrealistic opinions (e.g., [5]). However it has been argued that simply increasing people′s information may not change this scenario [68,69], given that increased knowledge of animal agriculture in general [24], specific practices [46,70], or having visited a farm [14] appears to increase, rather than decrease, concerns regarding the welfare of agricultural animals. 

This study utilized the online survey tool Amazon’s Mechanical Turk (MTurk), which enabled quick access to participants who are at least as diverse and more representative of non-college populations previously used in typical Internet and traditional sampling regimes [17]. These authors also provided evidence that despite the financial compensation, payment does not appear to affect data quality. We also recognize that our results may have been influenced to some extent by respondents’ social desirability bias (i.e., respondents appearing more sensitive to issues than they are in reality), however, it has been shown that online self-administered surveys minimize this effect (e.g., [71]).

One objective of this study was to assess whether there would be differences in response patterns by adopting the expression “pig farming” or “pork farming”, considering that maybe the latter could dispel concerns, related to the animals as sentient beings. In a survey conducted in Denmark, Lassen et al. [15] found that, when talking about pork, the interviewees characteristically limited their remarks to price and what can be described as the material quality of pork. However, in our study we noted no clear differences in the responses between both cohorts; respondents of both cohorts understood a pig or pork farm as a commercial unit that produced pork for human consumption, resulting in similar concerns raised by both cohorts. Also of interest, 14% of our participants indicated that they had some involvement with the pork industry; however, they raised similar issues such as space, humane care, access to the outdoors, and animal welfare as those with no involvement in pork production. However, equally interesting was that those with no involvement in pork production also raised issues normally viewed to be of concern by those working in the industry, such as production and economics.

Lastly, this study, based on a relatively small, convenience sample of participants living in 30 of the 50 U.S. states, and as such does not represent the views of the American society. When compared with a representative sample of Americans, our sample contained primarily respondents who are 18–35 years of age, referred to by many as the millennial generation. The technology used for sampling itself may have resulted in a skewed population response, favoring younger or more technologically savvy respondents, a point, which should be raised and discussed. However, this generation arguably represents the up incoming group of Americans who will be the primary purchasers of food, and that arguably will be influential in determining policies associated with food animal agriculture.

This study made use of a single open-ended question, which allows us to use well established social science methods to analyse the qualitative responses and to identify the connection of themes at an individual participant level. This type of qualitative research can provide important insights into which factors about pig/pork farms are the most important to the general public, as well as to identify potential areas of concern. Two logical follow up studies from this one would be firstly, to do in-depth interviews to further understand the beliefs and values of citizens, and secondly, to identify the proportions of individuals who have similar attitudes—this type of question would be better suited to a quantitative approach that uses a larger, representative sample. Our results can be useful and arguably important to consider when developing a quantitative survey targeting a representative sample, with full stratification across states, ages, and income groups, etc.

## 5. Conclusions

Participants expressed concerns about animal welfare that were both moral and ethical in nature, but also appeared supportive of swine production systems and practices that promote high sanitary and food safety standards. In general, they also desired rearing conditions for pigs that are associated with high meat quality, findings that are similar from studies done in many other countries. In summary, we encourage the U.S. swine industry to continually reflect on the types of practices they use, particularly in relation to the restriction of movement, when caring for their animals as practices that fail to resonate with societal values could risk the long terms sustainability of their industry.

## Figures and Tables

**Table 1:** Emergent themes in response to the question, “What do you consider to be an ideal pig/pork farm and why are these characteristics important to you?”.

Theme	Pork Farm *n* = 105	Pig Farm *n* = 94	Total *n* = 199
	%	%	%
Animal welfare	72	77	74
Business operation	44	43	44
Naturalness	25	30	27
Ethical considerations	25	24	25
Use of antibiotics, hormones, and “chemical residues”	22	15	19
Environment	10	16	13

**Table 2:** Participant demographics of the cohorts that participated in an online survey where they were asked to respond to the question, “What do you consider to be an ideal pig/pork farm and why are these characteristics important to you?”.

Demographics	Variable	Pig Farm (%)	Pork Farm (%)
		*n* = 94	*n* = 105
Sex	Male	57.4	65.7
	Female	42.6	34.3
Age	18–24	25.5	18.1
	25–34	39.4	47.6
	35–44	19.2	18.1
	>45	16.0	16.2
Level of education	Some high school	1.1	0.0
	High school graduate or equivalent	5.3	13.3
	Trade or vocational degree	0.0	1.0
	Some college	22.3	26.7
	Associate degree	12.8	10.5
	Bachelor’s degree	50.0	37.1
	Graduate or professional degree	8.5	11.4
Area of residence	Urban	31.9	23.8
	Rural	25.5	27.6
	Suburban	42.6	48.6
Time with household pet	Never	10.6	9.5
	<1 year	3.2	2.9
	1–5 years	16.0	15.2
	>5 years	70.2	72.4
Familiarity with Pig/Pork Farming	Very familiar	1.1	4.8
	Somewhat familiar	45.7	42.9
	Not familiar	53.2	52.4
Involvement with Pig/Pork farming	Farmer	1.1	2.9
	Veterinarian	0.0	1.0
	Agronomist or Animal Scientist	0.0	1.0
	Agricultural Science	1.1	1.9
	Pig/Pork Industry Professional	0.0	1.0
	Animal Advocate	9.6	7.6
	Not Involved	86.2	85.7
	Other	3.2	4.8
Vegetarian or vegan	Yes	7.4	7.6
	No	92.6	92.4

## Supplementary Materials

The following are available online at www.mdpi.com/2076-2615/7/8/64/s1. A copy of the survey is available as supplementary material.
