# Supplementary material for: American Citizens’ Views of an Ideal Pig Farm
Source: Animals (Basel). 2017 Aug 22;7(8):64. doi: 10.3390/ani7080064 (PMC5575576; doi:10.3390/ani7080064)
Supplement: Supplementary file 1 [file animals-07-00064-s001.pdf]

IDEAL PIG/PORK FARM SURVEY
(Sato et al.)

SINGLE OPEN ENDED QUESTION RANDOMIZED ACROSS PARTICIPANTS

What do you consider to be an ideal pig farm and why are these characteristics important to you?

OR

What do you consider to be an ideal pork farm and why are these characteristics important to you?

FOLLOWUP DEMOGRAPHIC QUESTIONS

1. **Sex**
( ) Female
( ) Male

2. **How old are you? (open question) ____________**

3. **Which best describes your level of education?**
( ) Some High School
( ) High School Graduate or Equivalent
( ) Trade or Vocational Degree
( ) Some College
( ) Associate Degree
( ) Bachelor's Degree
( ) Graduate or Professional Degree

4. **In which country were you born? ___________________**

5. **In which state do you currently live? _________________**

6. **Have you lived in the United States for the majority of your life?**
( ) Yes
( ) No

7. **Which best describes where you have lived most of your life?**
( ) Rural
( ) Suburban
( ) Urban

8. **How many years have you lived with a household pet?**
( ) I have never lived with a pet
( ) Less than a year
( ) 1 - 5 years
( ) 5 or more years

9. Please indicate your familiarity with pig/pork farming.
(   ) Not familiar
(   ) Somewhat familiar
(   ) Very familiar

10. Please indicate your involvement in the pig/pork industry.
(   ) Farmer
(   ) Veterinarian
(   ) Agronomist or Animal Scientist
(   ) Student or Teacher in Agricultural Science
(   ) Pig/Pork Industry Professional
(   ) Animal Advocate
(   ) Not Involved with Pig/Pork Production
(   ) Other, please specify: ___________

11. Are you vegetarian or vegan?
(   ) Yes
(   ) No
